# Towards the prevention of childhood leukemia

**DOI:** 10.18632/oncoscience.553

**Published:** 2022-04-21

**Authors:** Kim E. Nichols, Isidro Sánchez-García

**Keywords:** leukemia, infection, murine models, genetic susceptibility, prevention

B-cell acute lymphoblastic leukemia (B-ALL) is the most common childhood cancer and leading cause of pediatric cancer death. In childhood B-ALL, a mutation (hereditary or *de novo*) leads to appearance of preleukemic cells that are capable of normal lymphoid differentiation; however, upon acquisition of one or more second hit mutations, these preleukemic cells transform into full-blown leukemic blasts. While identification of the specific events that trigger the malignant evolution of preleukemic cells has remained elusive in humans, it has long been hypothesized that (delayed) exposure to infection promotes an immune response that then spurs the acquisition of additional genetic lesions [1].

Recently, independent studies using different genetically predisposed mice have demonstrated the occurrence of such an infection-triggered leukemogenic mechanism, collectively showing that several types of stress in the immune system can promote clonal evolution of preleukemic cells in a significant proportion of mice [2–4]. Interestingly, the immune stress does not act by selecting a preleukemic clone that already harbors the second hit; on the contrary, the infection acts by promoting acquisition of the second hit itself, therefore leading to full-blown B-ALL [1]. Together, these observations support the idea that by eliminating preleukemic cells, childhood B-ALL might be preventable [1–5]. Nevertheless, it has remained unclear how to target pre-leukemic cells as a means to prevent the development of B-ALL.

To address this question, we took advantage of the *Pax5*^+/−^ mice [2, 4]. Similar to children who harbor heterzygous germline *PAX5* mutations, B-ALL develops in up to 25% of *Pax5*^+/−^ mice, but only when these animals experience an immune stress, such as exposure to infection [2]. The leukemias that develop in this model acquire various types of second hit mutations which resemble those observed in human B-ALL, including activating mutations affecting the Janus Kinases (JAKs) [2]. We previously observed that pro-B cells in *Pax5*^+/−^ mice are particularly dependent on the cytokine interleukin-7 (IL-7) for their survival, and that blocking IL-7-induced signaling using the JAK1/2 inhibitor ruxolitnib led to increased cell death *in vitro* [2]. Based on these findings, we used *Pax5*^+/−^ mice to explore whether treatment with ruxolitinib early in life might eliminate preleukemic cells *in vivo* and thus prevent B-ALL development [6]. Towards this end, we first established the optimal dosing scheme by performing pharmacokinetic studies in mice treated with ruxolitinib-containing chow. Consistent with their heightened dependence on IL-7 for survival *in vitro*, we observed that ruxolitinib treatment preferentially killed *Pax5*^+/−^ versus wild-type (WT) B-cell progenitors *in vivo*. Next, we treated *Pax5*^+/−^ and WT mice with vehicle or ruxolitinib-containing chow for 14 or 28 days, beginning immediately after the mice were moved from an specific–pathogen-free facility (the one into which they were born and weaned) into one where they were exposed to common mouse pathogens. We monitored mice over time and compared the B-ALL incidence within the various cohorts. Notably, mice fed with ruxolitinib-containing chow for 28 but not 14 days exhibited a significant 90% reduction in B-ALL development compared to mice fed with control chow.

**Figure 1:**
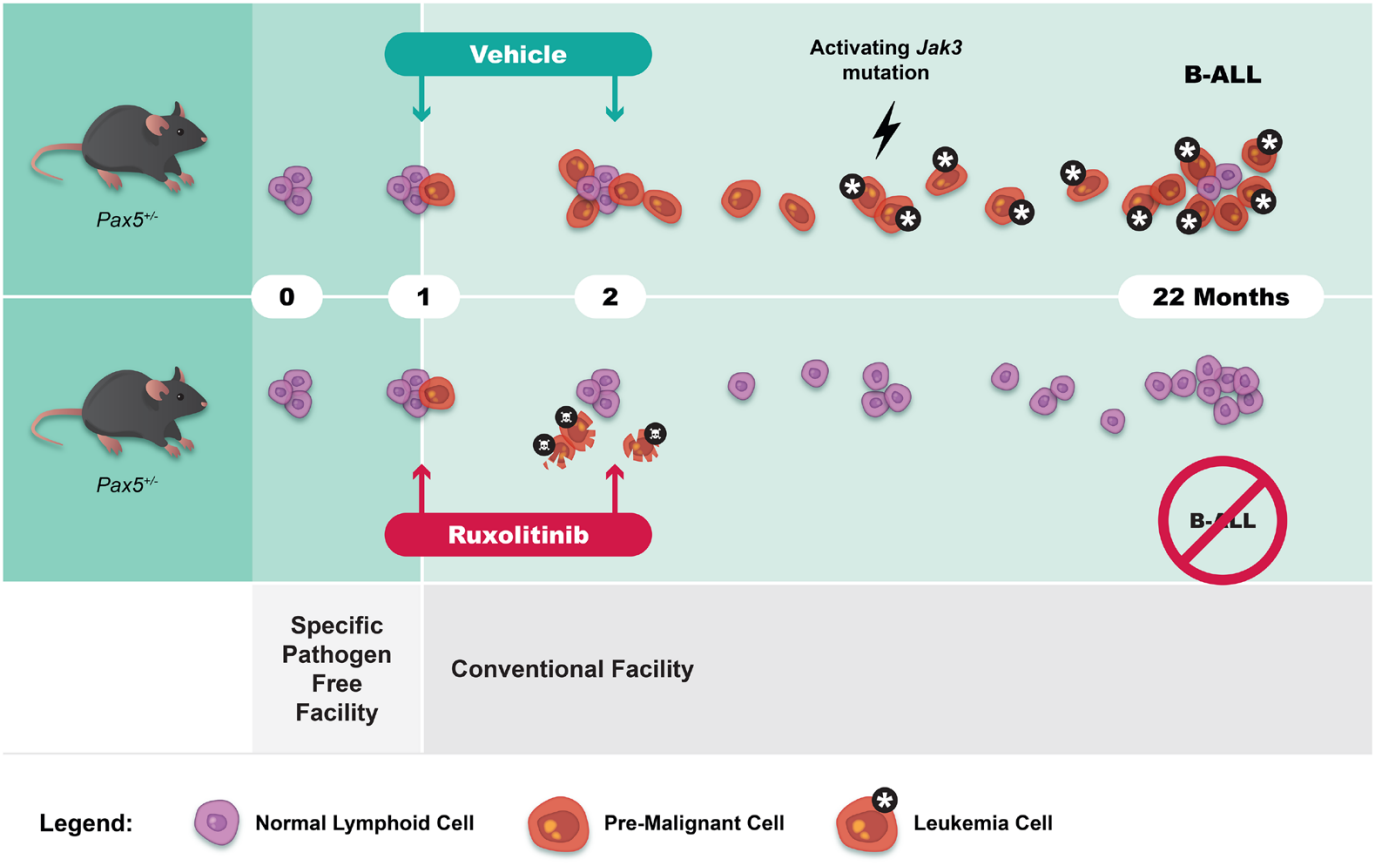
An accessible window of opportunity in early postnatal life during which B-ALL prevention might be possible and effective. In the majority of *Pax5*^+/−^ mice, preleukemic progenitor B cells allow normal B cell development under most circumstances (violet colored cells). However, immune stressors, such as transfer to a conventional mouse facility that contains common mouse pathogens, triggers progression towards B-ALL (rust colored cells) through the accumulation of secondary mutations affecting the JAK/STAT pathway. Transient treatment of *Pax5*^+/−^ mice with the JAK1/2 inhibitor ruxolitinib significantly reduces the risk of leukemia development.

This severe reduction in B-ALL incidence after 28 days of ruxolitinib treatment could conceivably be caused by the elimination of *Pax5*^+/−^ B-cell progenitors harboring second hit leukemogenic mutations in the JAK/STAT pathway (either acquired during the 4-week treatment period, or present before); however, this seems unlikely, since previous ultradeep sequencing studies have shown that somatic *Jak* mutations only appear in *Pax5*^+/−^ mice several months later once the animals already present with full-blown B-ALL [2]. By showing that *Pax5*^+/−^ mice carrying an active *Jak3*^V670A^ transgene develop B-ALL immediately in the absence of pathogen exposure, we conclude that the effect of ruxolitinib is likely due to the elimination of “at risk” pro-B cells in which the second hit will later arise (Figure 1). Taken together, our results demonstrate that JAK-STAT inhibition using ruxolitinib prevents *Pax5*-associated B-ALL in mice and suggest that a similar strategy might be useful for children at risk of developing B-ALL in the context of *PAX5* or other predisposing germline mutations. Finally, because the protective effect of ruxolitinib occurred following transient treatment of *Pax5*^+/−^ mice, it remains possible that B-ALL prevention in children might not require life-long but rather shorter-term treatment during a defined time period.

As the presence of a latent premalignant cell is a common characteristic of many types of human leukemias, as well as other cancers [7], this study offers a general proof-of-principle for the development of similar preventive strategies for other cancers. Despite these findings, there are several questions that remain unanswered. For example, why do most genetically-predisposed mice (and children carrying predisposing mutations) remain healthy and never develop leukemia? Why and how do extrinsic factors, including immune stressors such as infection, facilitate the clonal evolution of preleukemic cells? The exact nature, timing, mechanism, and sequence of events resulting in the appearance of B-ALL warrants further investigation.
